# Efficient online detection device and method for cottonseed breakage based on Light-YOLO

**DOI:** 10.3389/fpls.2024.1418224

**Published:** 2024-08-09

**Authors:** Hongzhou Zhang, Qingxu Li, Zhenwei Luo

**Affiliations:** ^1^ College of Mechanical and Electrical Engineering, Tarim University, Alar, China; ^2^ Institute of Cotton Engineering, Anhui University of Finance & Economics, Bengbu, China

**Keywords:** cottonseed, breakage, YOLOV8m, Light-YOLO, online detection

## Abstract

High-quality cottonseed is essential for successful cotton production. The integrity of cottonseed hulls plays a pivotal role in fostering the germination and growth of cotton plants. Consequently, it is crucial to eliminate broken cottonseeds before the cotton planting process. Regrettably, there is a lack of rapid and cost-effective methods for detecting broken cottonseed at this critical stage. To address this issue, this study developed a dual-camera system for acquiring front and back images of multiple cottonseeds. Based on this system, we designed the hardware, software, and control systems required for the online detection of cottonseed breakage. Moreover, to enhance the performance of cottonseed breakage detection, we improved the backbone and YOLO head of YOLOV8m by incorporating MobileOne-block and GhostConv, resulting in Light-YOLO. Light-YOLO achieved detection metrics of 93.8% precision, 97.2% recall, 98.9% mAP50, and 96.1% accuracy for detecting cottonseed breakage, with a compact model size of 41.3 MB. In comparison, YOLOV8m reported metrics of 93.7% precision, 95.0% recall, 99.0% mAP50, and 95.2% accuracy, with a larger model size of 49.6 MB. To further validate the performance of the online detection device and Light-YOLO, this study conducted an online validation experiment, which resulted in a detection accuracy of 86.7% for cottonseed breakage information. The results demonstrate that Light-YOLO exhibits superior detection performance and faster speed compared to YOLOV8m, confirming the feasibility of the online detection technology proposed in this study. This technology provides an effective method for sorting broken cottonseeds.

## 1 Introduction

Cotton plays a pivotal role in daily life, closely intertwined with essential sectors such as clothing, medicine, and agriculture. The significance of cotton production extends beyond mere economic implications; it is paramount in ensuring the sustenance and development of human life (Zhang et al., 2022). The quality of cottonseeds emerges as a pivotal determinant in the cotton production process. Intact cottonseed hulls not only mitigate nutrient loss during storage but also act as a barrier against microbial infestation, thereby creating favorable conditions for the germination and growth of cotton (Liu et al., 2022). Despite the advantages of intact cottonseed hulls in facilitating the germination and growth of cotton, the series of processes such as ginning, fluffing, and polishing in cottonseed production lead to substantial breakage (Tostes et al., 2023). Broken cottonseeds exhibit reduced germination rates and diminished vitality of cotton seedlings (Zhang et al., 2022). If not promptly removed, they can lead to widespread replanting in cotton fields, thereby increasing the overall cost of cotton cultivation. Consequently, the removal of broken cottonseeds before planting becomes imperative. Currently, in Xinjiang, the predominant method for detecting broken cottonseeds involves iron powder adsorption followed by magnetic separation. However, with an annual cotton planting area exceeding 2500 hectares in Xinjiang, this method leads to considerable resource wastage (Lu et al., 2022). Furthermore, this sorting method falls short of the desired effectiveness, resulting in a significant portion of cotton requiring replanting (Li et al., 2023a). Hence, there is an urgent demand for a rapid and cost-effective solution for detecting and sorting broken cottonseeds.

To address the challenge of detecting broken cottonseeds, researchers have explored various technical approaches, including machine vision, color sorting, and spectral imaging technology. In the domain of color sorting technology, Shuhua et al. (2015) utilized dual CCD cameras to capture image information of both broken and intact cottonseeds. Their method distinguished these types by differences in pixel area, achieving a detection accuracy of 97.8%. In spectral imaging, Gao et al. (2018) achieved breakage recognition of cottonseeds by capturing hyperspectral data in the range of 874~1734 nm. By combining the image and spectral features of cottonseeds, they attained a recognition accuracy of 91.50%. In the field of machine vision, Wang et al. (2023) employed a high-speed camera to capture images of falling cottonseeds and integrated it with YOLOv5S, resulting in a 99% accuracy in recognizing broken and moldy cottonseeds. Similarly, Liu et al. (2022) utilized YOLOv5S for the detection of broken cottonseeds in images of multiple cottonseeds, achieving an accuracy of 92.4%. Additionally, Du et al. (2023) utilized the ResNet50 network with an added attention mechanism to classify both broken and intact cottonseed images, achieving an accuracy of 97.23%. Furthermore, Zhang et al. (2022) employed air-coupled ultrasound with a sound-to-image encoding technique for detecting slightly cracked cottonseeds, achieving a recognition accuracy of 90.7%. **Table 1** provides a detailed and systematic comparison of the strengths and weaknesses identified in the previously mentioned studies. Despite these advancements, current methods still face several technical gaps. The iron powder adsorption and magnetic separation method used in Xinjiang is resource-intensive and inefficient. Although accurate, color sorting and spectral imaging are costly. Furthermore, most existing studies focus on detecting single cottonseeds and cannot simultaneously process multiple cottonseeds, which is crucial for large-scale operations. Only Liu et al. (2022) and Gao et al. (2018) achieved simultaneous detection of multiple cottonseeds; however, their methods only detected one side of the cottonseeds. Other methods have concentrated solely on single cottonseed detection, which does not fully meet the practical needs of the cottonseed industry.

**Table 1:** Summary of research.

Technology	Study	S/M	Detection Content	Results	Disadvantages
Color sorting	Shuhua et al., 2015	S	Broken cottonseeds	97.8%	High cost
Machine vision	Wang et al., 2023	S	Broken and moldy cottonseeds	99%	Low detection efficiency
	Liu et al., 2022	M	Broken cottonseeds	92.4%	Only detect single-sided cracks in cottonseeds, unable to adapt to actual production conditions
	Du et al., 2023	S	Broken cottonseeds	97.23%	Only detect single-sided cracks in cottonseeds
Spectral imaging	Gao et al., 2018	M	Slightly cracked cottonseeds	91.5%	High cost, only detect single-sided cracks in cottonseeds
Others	Zhang et al., 2022	S	Slightly cracked cottonseeds	90.7%	Difficult to apply in production

S, Single cottonseed; M, Multiple cottonseeds.

The preceding study demonstrates that machine vision technology is proficient in detecting cottonseed breakage, offering notable advantages in cost-effectiveness and stability over hyperspectral imaging, high-speed cameras, and air-coupled ultrasound devices. Moreover, machine vision systems exhibit the capability to detect multiple cottonseeds simultaneously. Notably, among the studies discussed earlier, only Liu et al. (2022) achieved simultaneous detection of multiple cottonseeds, whereas other studies focused solely on single cottonseeds. Simultaneously achieving rapid detection of multiple cottonseeds breakage would not only enhance efficiency but also align more closely with the practical requirements of the cottonseed industry. Achieving simultaneous detection of multiple cottonseeds typically necessitates the application of object detection algorithms (Zhao et al., 2020). Recently, object detection has witnessed numerous successful applications in the field of seed research (Zhou et al., 2023). Examples include the identification of barley seed varieties (Shi et al., 2022), assessment of rice seed vitality (Qiao et al., 2023), measurement of rice seed size (Zhao et al., 2023), and detection of defective lotus seeds (Lu et al., 2022).

In conclusion, this study comprehensively reviews previous research on cottonseed breakage detection, extracting valuable insights and leveraging their respective strengths. To enable simultaneous detection of breakage on both sides of cottonseeds and to efficiently and rapidly detect multiple cottonseeds at once, thus providing an effective solution and technology for the cottonseed industry’s broken cottonseed detection, this study proposes constructing an image acquisition device capable of capturing images of both sides of 15 cottonseeds simultaneously. The collected cottonseed images are enhanced using data augmentation algorithms, and the YOLOV8 algorithm is employed to detect broken cottonseeds in these images. To improve detection speed and accuracy, we introduce modules like MobileOne and GhostConv to develop a Light-YOLO, which is based on YOLOV8. Finally, to facilitate the rapid application of this method in practical production, a software, hardware, and control system for the online detection of broken cottonseeds is developed and the online detection device is validated. Compared to existing studies on broken cottonseed detection, which are either limited to laboratory settings or involve high detection costs, the proposed methods and techniques in this study offer the following advantages: (1) detection of damage on both sides of the cottonseeds; (2) simultaneous detection of 15 cottonseeds, resulting in high detection efficiency; (3) direct application of the techniques and methods in devices, enabling these technologies to address practical production issues.

## 2 Materials and methods

### 2.1 Sample preparation

**Figure 1:**
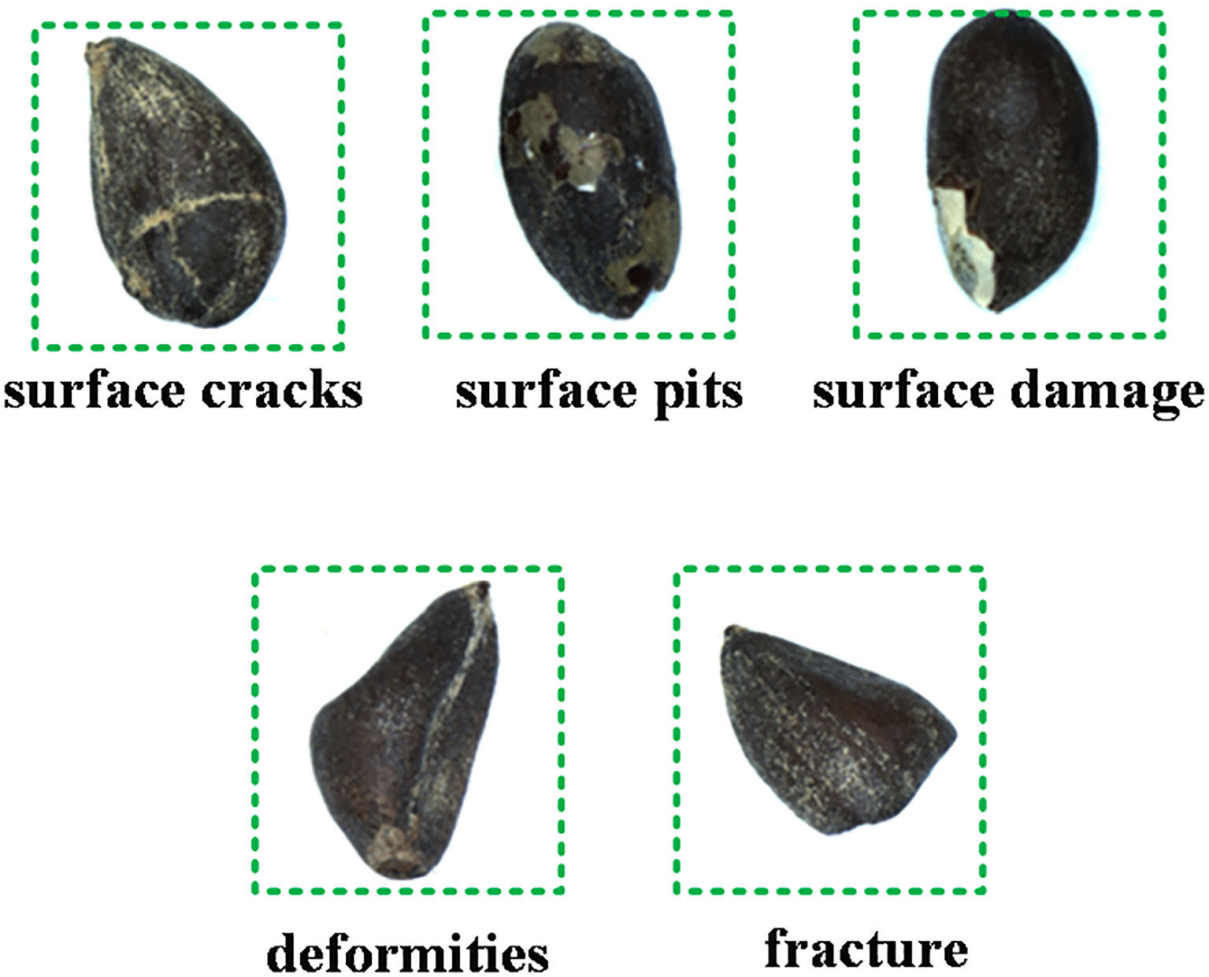
Types of broken cottonseeds.

We sourced the cottonseeds for this study from Xinjiang Tahe Seed Company, selecting a total of 3120 seeds, consisting of 1424 broken seeds and 1696 intact seeds. The specific variety of cottonseed used was Xinhai-63. All cottonseed surfaces underwent corrosion treatment with dilute sulfuric acid, resulting in smooth cottonseeds devoid of cotton fibers. To maintain randomness and systematic organization, this study assigned unique sequential numbers to each cottonseed and divided them into 208 groups of 15 seeds each. The broken cottonseed types within the test material selected for this study predominantly consisted of seeds exhibiting defects such as surface cracks, pits, damage, deformities, and fractures. These defects were often associated with the exposure of white seed flesh, as illustrated in **Figure 1**. In comparison to intact cottonseeds, these flawed cottonseeds are uniformly categorized as broken cottonseeds.

### 2.2 Image acquisition system

**Figure 2:**
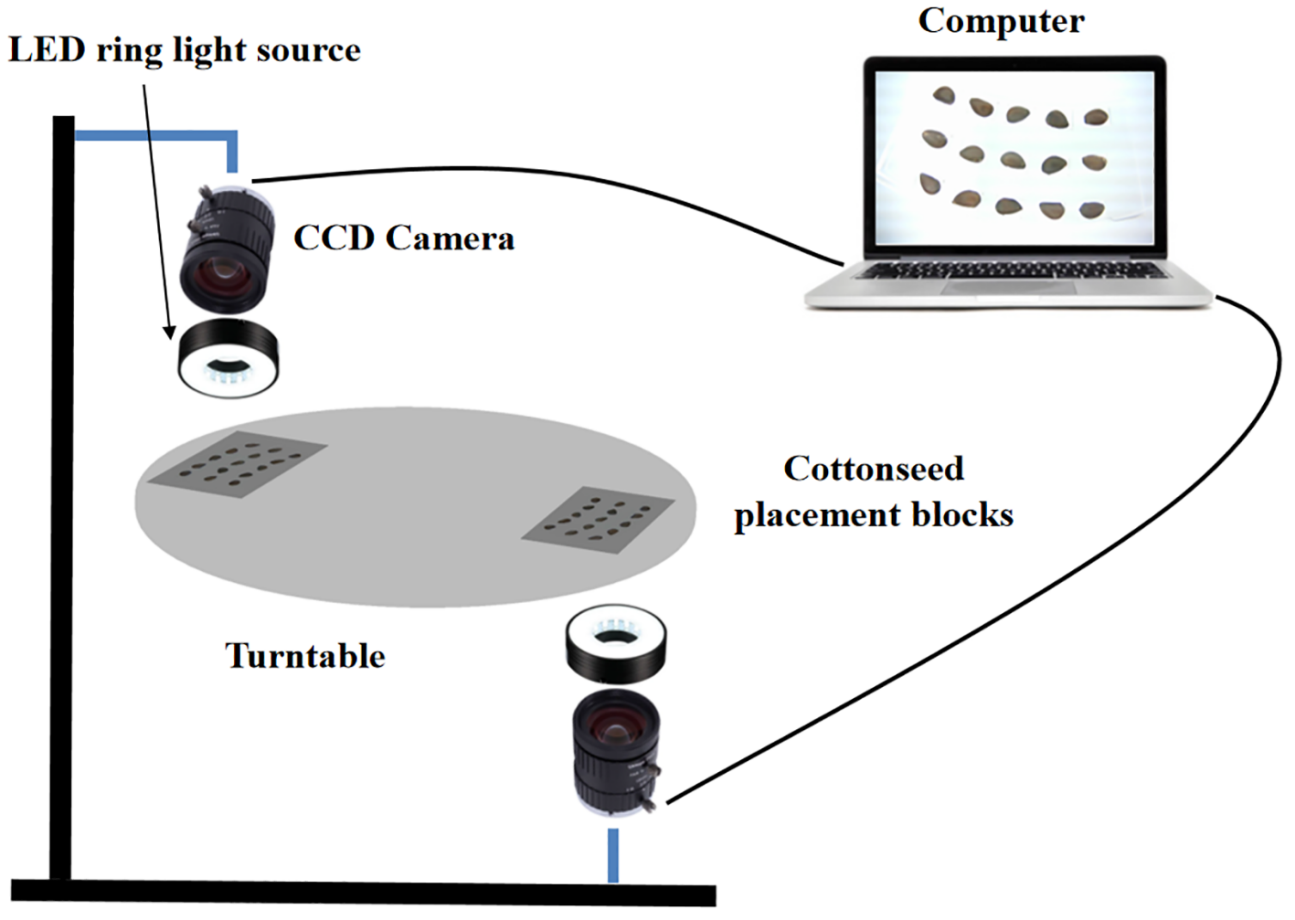
Schematic diagram of the cottonseed image acquisition system.

The image acquisition system is primarily composed of two CCD cameras, two LED ring light sources, a computer, a turntable, and a cottonseed placement module, as depicted in **Figure 2**. The utilization of two CCD cameras aims to capture information from both the front and back sides of the broken cottonseed. The camera model employed is the Green Vision Forest USB1080P, featuring an adjustable resolution of 1280 pixels × 720 pixels and an exposure degree of -9. The lens has a 5 ~ 50 mm zoom capability and utilizes an F1.4 industrial lens with the largest aperture. The LED ring light sources serve to mitigate the impact of ambient light on the cottonseed images, each having a power of 20W LED cool white light. The cottonseed placement platform, with a diameter of 600 mm, is made of lightweight aluminum and is designed for the transportation of cottonseeds used to simulate the image acquisition conditions of cottonseeds in the subsequent design of the device. The cottonseed placement module on the turntable is constructed using acrylic panels and is comprised of 15 cottonseed grooves. Each groove measures 10 mm in length, 6 mm in width, and 5 mm in depth. Arranging the grooves in a 3×5 grid optimizes imaging with the camera positioned beneath the seed placement platform. This arrangement allows for the simultaneous capture of up to 15 images of the cottonseeds. To operate the system, place 15 cottonseeds in the cottonseed placement module. Rotate the turntable to move the slot directly under the first camera, then stop the turntable and capture the top image of the cottonseeds. Next, rotate the turntable to move the slot directly above the second camera, stop the turntable again, and capture the bottom image of the cottonseeds. This process completes the two-sided image detection of the 15 cottonseeds.

### 2.3 Dataset preparation

#### 2.3.1 Data annotation and augmentation

The image acquisition system collected data for 3120 cottonseeds. Since the system captures 15 cottonseeds at a time and records both front and back images, we obtained a total of 416 images. Each image includes 15 cottonseeds, creating a dataset of 6240 cottonseed images. To tailor the collected image data to the training specifications of the object detection algorithm, this study utilized the LabelImg annotation tool. Based on the sample collection number and the actual label indicating whether the cottonseed is broken or intact, the broken and intact cottonseeds in the images are manually annotated. The annotations categorize intact cotton seeds as “intact” and broken cotton seeds as “broken”. Afterward, the 416 multiple cottonseeds images were partitioned into a training set, a test set, and a validation set. The training set was used to train the model parameters for distinguishing between intact and broken cottonseeds. It comprised 330 images containing a total of 4,950 cottonseeds, with 2,248 being broken and 2,702 being intact. The validation set, used for evaluating the model parameters during training, consisted of 42 images with 630 cottonseeds, including 318 broken and 312 intact cottonseeds. The test set was utilized to verify the model’s performance and included 44 images containing 660 cottonseeds, with 282 broken and 378 intact cottonseeds. The detailed distribution of the data is presented in **Table 2**.

**Table 2:** The distribution of data.

	Number of images	Intact	broken	Total
Training set	330	2702	2248	4950
Validation set	42	312	318	630
Test set	44	378	282	660
Total	416	3392	2848	6240

“Intact”, and “broken” represent the respective counts of each type of object in the dataset.

**Figure 3:**
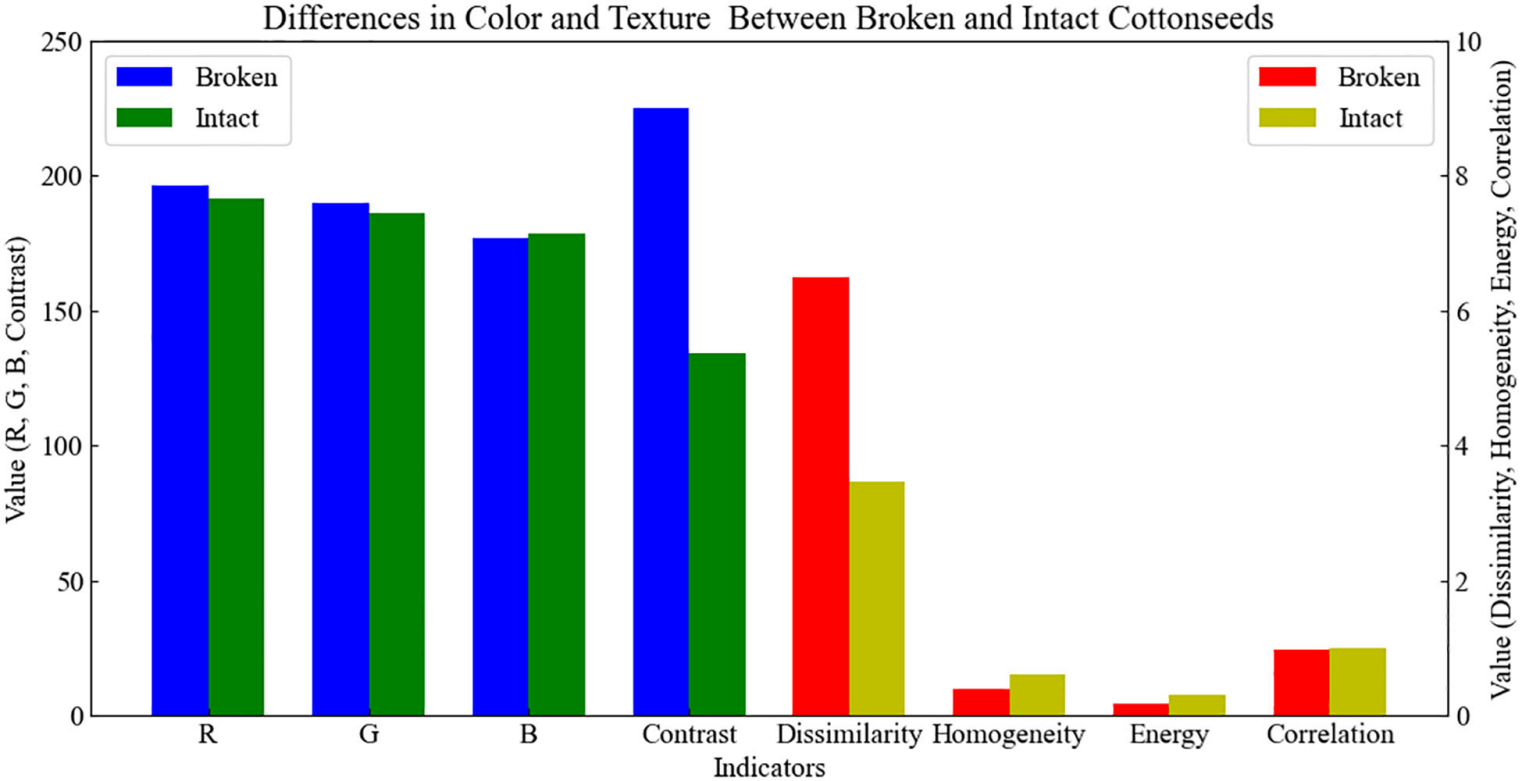
Differences in color and texture between broken and intact cottonseeds.

The greater the difference in image characteristics between intact and broken cottonseeds, the higher the detection accuracy of the subsequent detection model (Rangaswamy et al., 2022). To quantify the image differences between broken and intact cottonseeds, this study extracted color and texture features from each cottonseed in the dataset. The color features primarily include the mean pixel values of the R, G, and B color components. Texture features were extracted using the gray-level co-occurrence matrix (GLCM), including contrast, dissimilarity, homogeneity, energy, and correlation metrics. Subsequently, the mean values of R, G, and B color components, as well as contrast, dissimilarity, homogeneity, energy, and correlation for all broken and intact cottonseeds were calculated. The results, as shown in **Figure 3**, indicate significant differences in contrast, dissimilarity, homogeneity, and energy between broken and intact cottonseeds, while the differences in the mean pixel values of R, G, and B components and correlation are relatively small. This demonstrates that there are significant differences in image characteristics between intact and broken cottonseeds, allowing for the detection of broken cottonseeds based on these differences.

**Figure 4:**
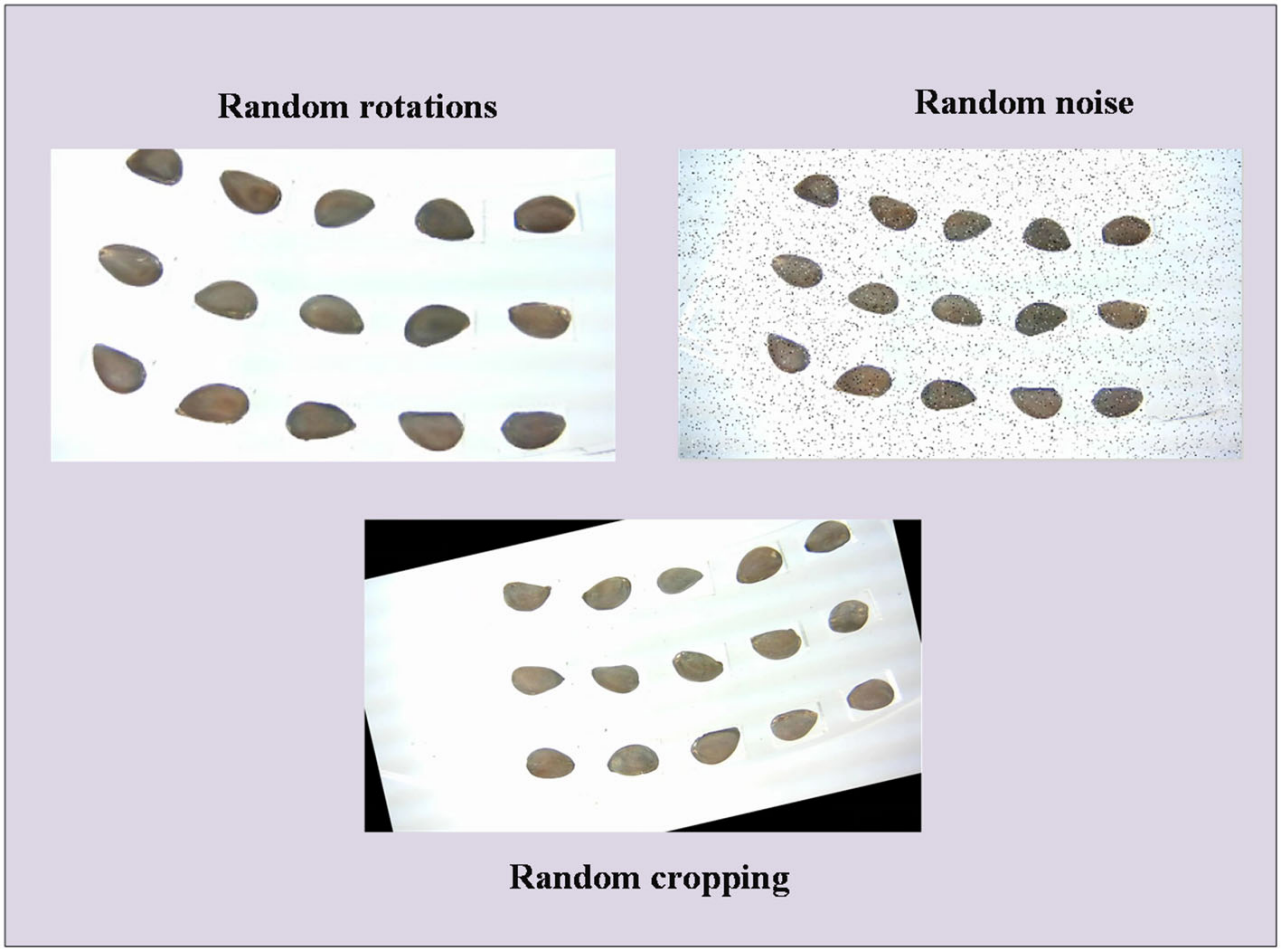
Data augmentation.

In theory, a larger dataset size with a more realistic sample distribution enhances the performance of trained deep learning models (Guo et al., 2023). To augment the dataset size, this study implements data augmentation on the training set. This involves random rotations, cropping, and the addition of perturbing noise to the images of multiple cottonseeds in the training set, as illustrated in **Figure 4**. After data augmentation, the training set comprises 990 images, encompassing a total of 14850 cottonseeds, maintaining the original proportion of intact and broken cottonseeds. The sample size in the validation and test sets remains unchanged. The dataset is ultimately formatted in YOLO format.

### 2.4 YOLOV8

#### 2.4.1 Input

YOLOV8 is a real-time detection algorithm known for its enhanced accuracy and rapid processing speed (Reis et al., 2023), widely used in industrial and agricultural applications (Wang et al., 2023; Yang et al., 2023). In this study, we utilized YOLOV8 to detect intact and broken cottonseeds within multiple seeds, to swiftly scale up the technology in the cottonseed industry due to its superior detection speed and accuracy. It comprises three components: input, backbone, and YOLO-Head. In the input layer of YOLOv8, it is imperative to uniformly resize the multiple cottonseeds image to 640 pixels × 640 pixels. Similar to YOLOV4 and YOLOV5, data preprocessing methods such as Mosaic and Mixup are employed for augmenting the input image. The distinction lies in the fact that the YOLOV8 input layer does not incorporate anchor frame technology, and data augmentation is disabled ten epochs before the conclusion of training.

#### 2.4.2 Backbone

The backbone of YOLOV8 plays a primary role in automatically extracting image features at various scales. It comprises CBS modules, C2F modules, and SPPF module. The CBS module is primarily responsible for computing image features and includes a convolutional layer (with a kernel size is 3×3), Batch Normalization, and a SiLU layer. The convolutional layer computes features, the Batch Normalization layer accelerates the network’s convergence speed, and the SiLU layer functions as an activation function using the SiLU function, implemented as shown in Formula 1. The incorporation of the CBS module notably improves the model’s detection accuracy, particularly in effectively discerning small targets (Rong et al., 2023).


(1) $$SiLU(x)=\frac{x}{1+e^{x}}$$

**Figure 5:**
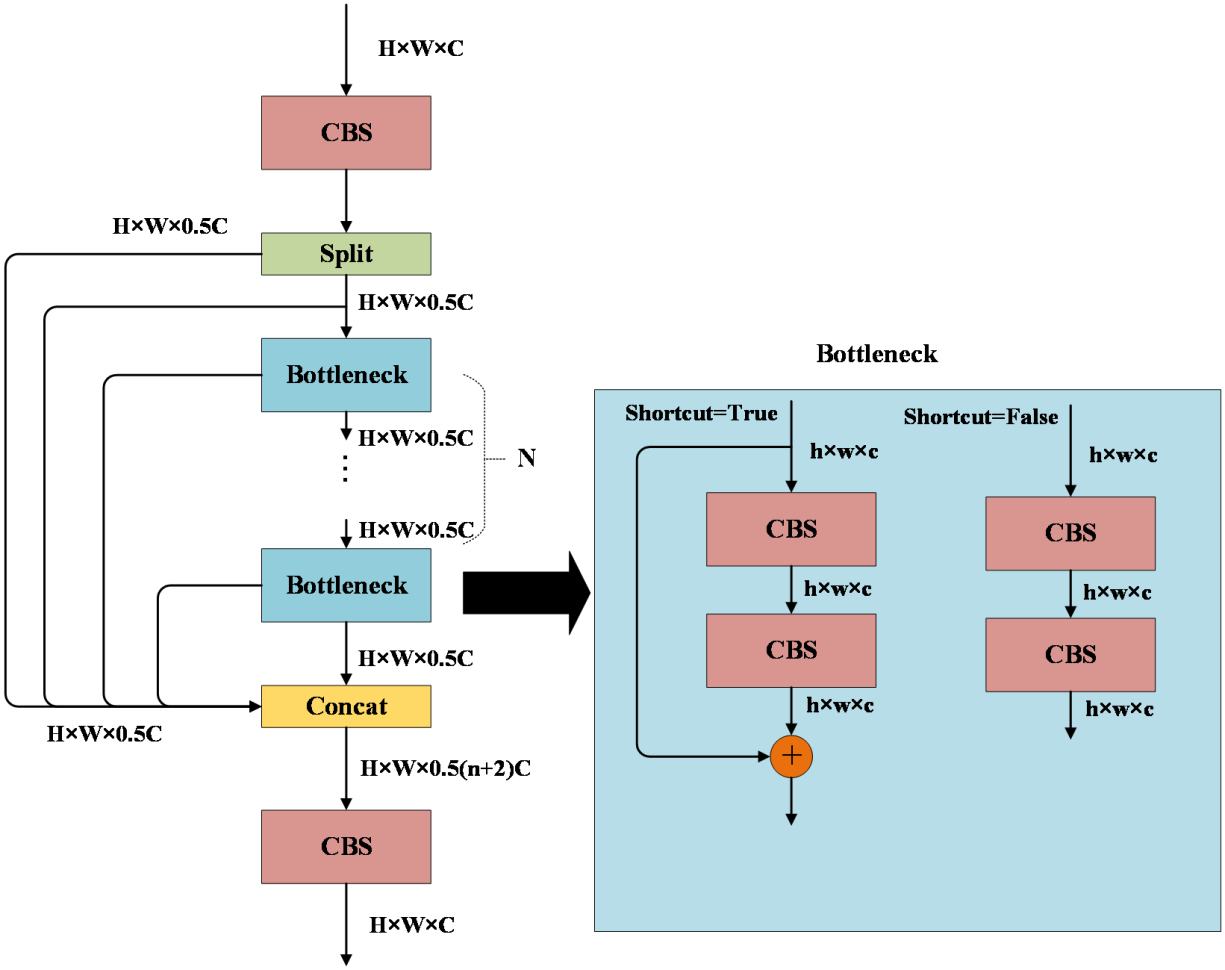
C2F module.

The C2F module is designed to introduce additional branches for enhancing tributaries during gradient backpropagation. It comprises 2 CBS modules with a 1×1 convolution kernel size and multiple Bottleneck modules, as depicted in **Figure 5**. Assuming the input image feature size is H×W×C, after the first CBS module, the feature size remains unchanged. Subsequently, it is split into 2 branches, each with a feature size of H×W×0.5C. One branch is directly inputted to Concat for fusion, while the other branch is inputted to the Bottleneck module. However, before inputting to the Bottleneck module, Concat fusion is also required. Each Bottleneck module is likewise inputted to Concat for fusion. If the C2F module contains n bottleneck modules, then the output feature size after Concat is H×W×0.5(n+2) C. The feature is then input to the second CBS module, and the final output image feature size is also H×W×C. In the Bottleneck module, which comprises two CBS modules, if the “shortcut” is set to True, it necessitates the addition of the input features and output features. The C2F module facilitates the fusion of low-level and high-level features in an image, enabling the model to leverage both detailed and semantic information. This enhancement contributes to improved accuracy and robustness in target detection (Zhang et al., 2023).

**Figure 6:**
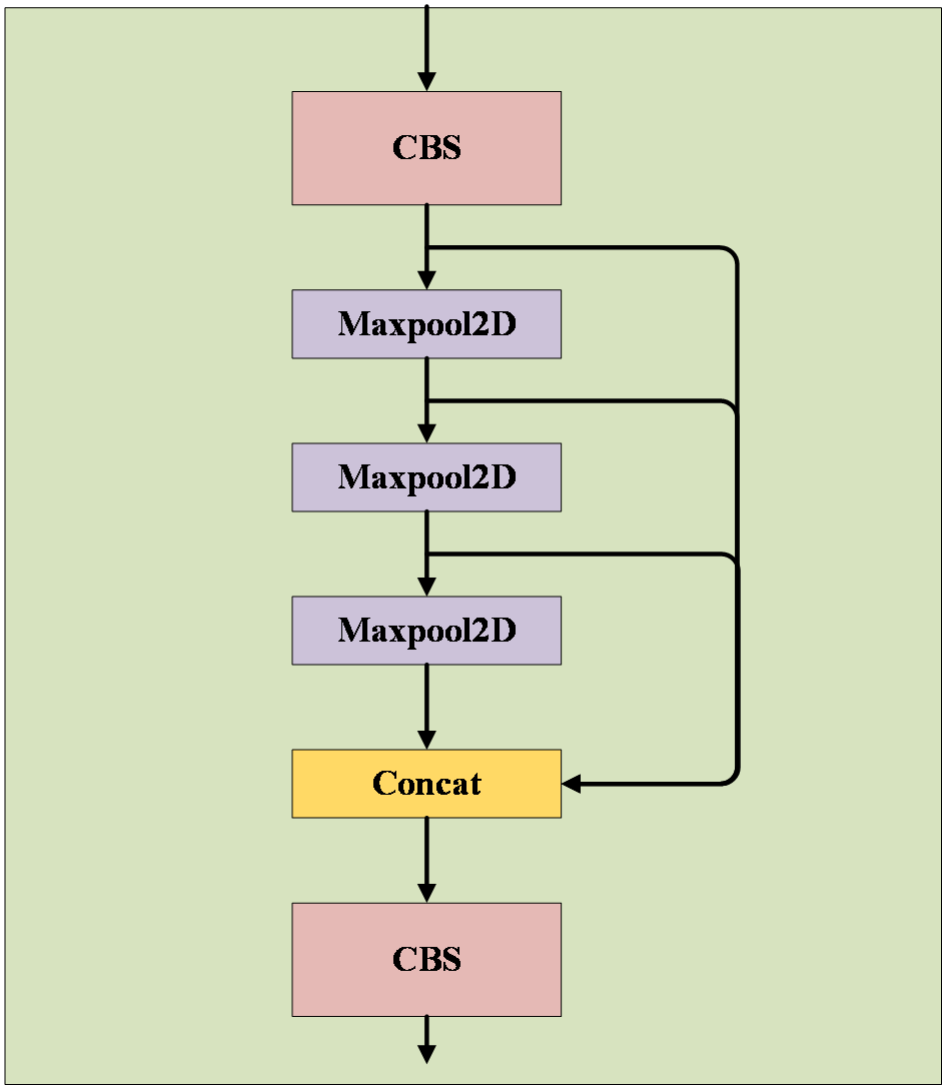
SPPF module.

The SPPF module adopts the concept of spatial pyramid pooling to combine feature maps at various scales, thereby creating more comprehensive feature representations. Comprising two CBS modules with a 1×1 convolution kernel size and three maximum pooling layers, as illustrated in **Figure 6**, these layers execute maximum pooling operations on feature maps at different scales to extract the most significant features from each scale. Subsequently, these essential features are concatenated to produce a fused feature map incorporating multi-scale information. The SPPF module’s advantage lies in its ability to enhance target detection accuracy through multi-scale feature fusion, particularly beneficial for targets with substantial size variations. Moreover, this module effectively mitigates issues related to image distortion caused by cropping and scaling operations on image regions. It also addresses the problem of redundant feature extraction in images by convolutional neural networks, thereby improving the speed of generating candidate frames and reducing computational costs.

#### 2.4.3 YOLO head

**Figure 7:**
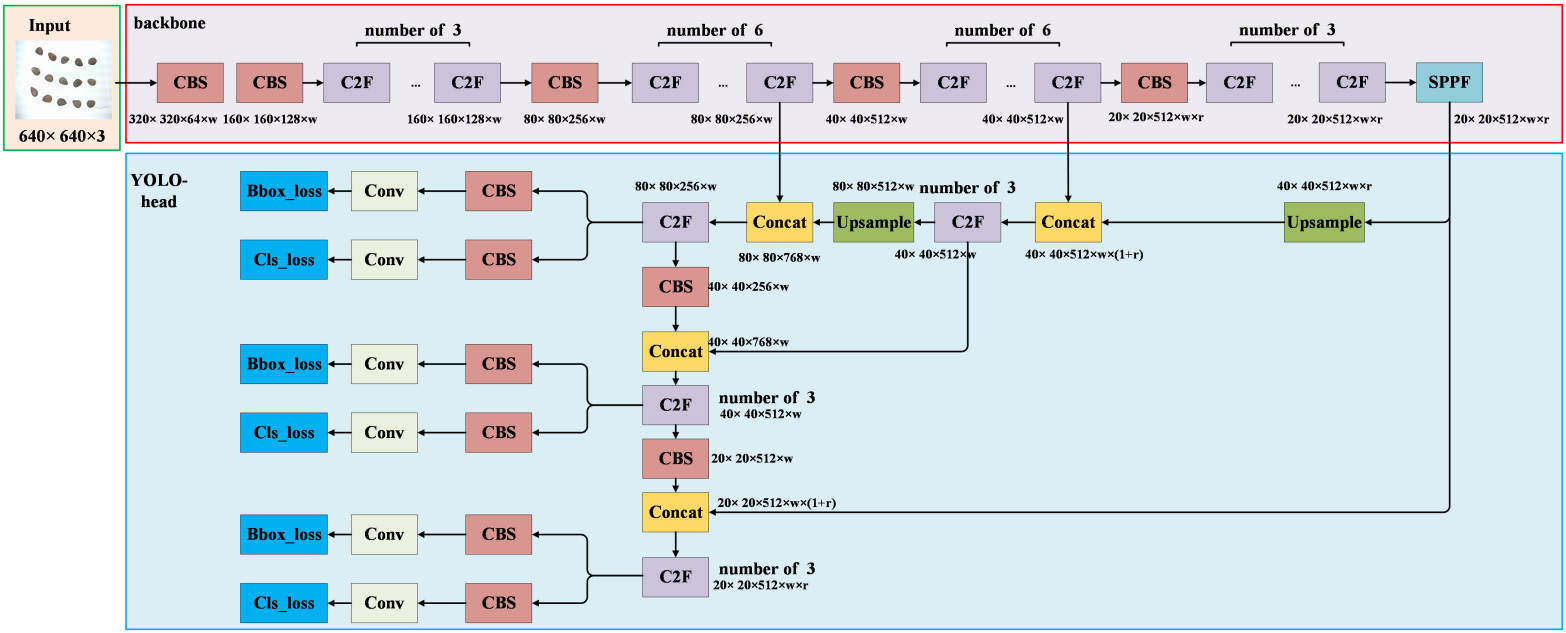
YOLOV8.

The network structure of YOLOV8 is illustrated in **Figure 7**. The YOLO head continues to employ the PAFPN (Path Aggregation Feature Pyramid Network) structure seen in previous YOLO versions. This structure enables the fusion of high-level and low-level features in the image, thereby enhancing the model’s detection performance for targets of varying sizes (Xu et al., 2021). Furthermore, the output section of the YOLO head adopts the Decoupled-Head structure, segregating the detection head for classification and localization information. The YOLO head encompasses the Upsample, C2F, CBS (with a 3×3 convolution kernel), and Conv modules (with a 1×1 convolution kernel). The Upsample module primarily performs upsampling operations on image features, facilitating the fusion of high-level and low-level features. The Conv module provides convolution operations for the output segment of the network. The C2F and CBS modules maintain consistency with their counterparts in the backbone. In the diagram, “w” and “r” represent the coefficients for the width and depth of the network, which categorize YOLOV8 into five versions: “n”, “s”, “m”, “l”, and “x”. Each version corresponds to a distinct number of model parameters. In this study, YOLOV8 was employed for the detection of intact and broken cottonseeds. The YOLO head outputs category information as “intact” and “broken”, along with position information represented by the coordinates of cottonseeds in the image.

#### 2.4.4 Loss function

The model training process necessitates the application of a loss function to quantify the disparity between the predicted and actual values of the model. YOLOV8’s loss function comprises classification loss and regression loss. The classification loss function is VFL (Varifocal Loss), while the regression loss is represented as CIoU Loss + DFL (Distribution Focal Loss). CIoU considers the similarity in center distance, overlap area, and aspect ratio between the predicted frame and the actual frame, as seen in Formulas 2–4, and is implemented using the following formulas:


(2) $$CIoU=IoU-[\frac{\rho^{2}(b,b^{gt})}{c^{2}}+\alpha v]$$

(3) $$v=\frac{4}{\pi^{2}}(\arctan\frac{w^{gt}}{h^{gt}}-\arctan\frac{w}{h})$$

(4) $$\alpha =\frac{v}{1-IoU+v}$$

where IoU represents the intersection over union ratio between the ground truth and the prediction box. ρ represents the Euclidean distance between the centroids of the ground truth and the prediction box. b represents the centroids of the prediction box. $b^{gt}$ represents the centroids of the ground truth. c represents the Euclidean distance between the prediction box and the ground truth’s diagonal. v represents the similarity of the prediction box to the aspect ratio of the ground truth. α represents a weight function. $w^{gt}$ , $h^{gt}$ represent the width and height of the ground truth. w , h represent the width and height of the prediction box.

The Varifocal Loss function addresses the issue of positive and negative sample imbalance through the application of an asymmetric weighting operation, achieved by the Formula 5:


(5) $$VFL(p,q)=\{\begin{array}{ll} -\alpha p^{\gamma }\log(1-p) & q=0\\ -q(q\log(p)+(1-q)\log(1-p)) & q>0 \end{array}$$

q represents the label, and q corresponds to the IoU value for positive samples, and vice versa when q is 0. The hyperparameter α governs the weighting of positive and negative samples. Additionally, γ is a hyperparameter that fine-tunes the weights of positive and negative samples, while p denotes the probability predicted by the model.

The Distribution Focal Loss function holds a distinct advantage in addressing target overlap, enabling the network to rapidly concentrate on the distribution of locations proximate to the target location. This is implemented with the Formula 6:


(6) $$DFL(S_{i},S_{i+1})=-((y_{i+1}-y)\log(S_{i})+(y-y_{i})\log(S_{i+1}))$$

Here, y represents the position information of the target, and $y_{i}$ , $y_{i+1}$ denote the neighboring intervals closest to the target y , $y_{i}<y<y_{i+1}$ . $S_{i}$ , $S_{i+1}$ represent the outputs of the Softmax function for the surrounding intervals closest to the target y .

### 2.5 Light-YOLO

#### 2.5.1 MobileOne

**Figure 8:**
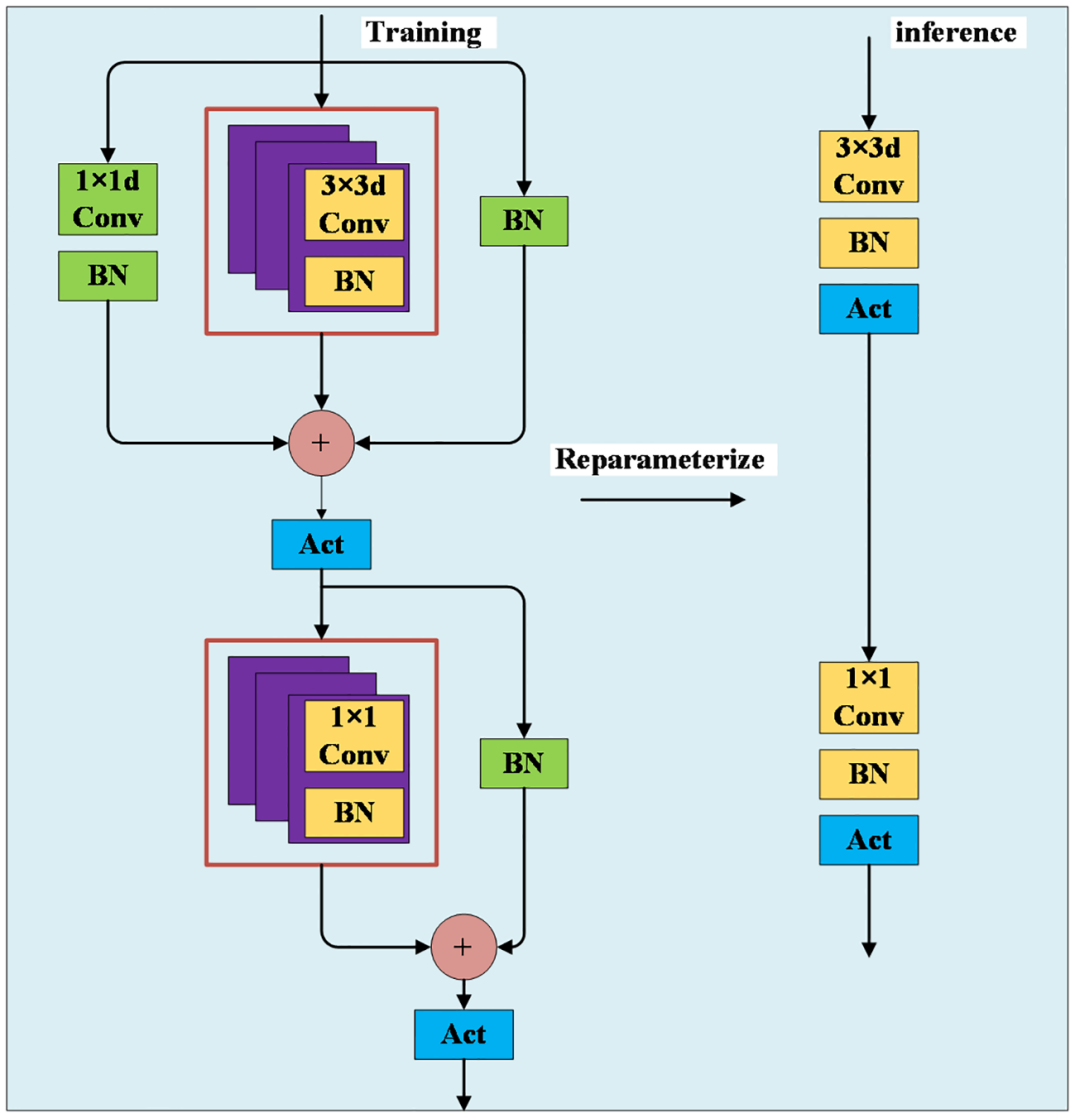
MobileOne-block.

The MobileOne network is a feature extraction network specifically designed for mobile applications, offering the dual advantages of speed and accuracy (Vasu et al., 2023). Traditional feature learning models, which include convolution, batch normalization (BN), and activation functions, often face degradation challenges as network depth increases. MobileOne addresses this issue by introducing a novel approach that uses three parallel branches. Its innovative design incorporates the concept of reparameterization, employing distinct network structures during the training and inference phases. During the training phase, a more complex structure is utilized to enhance learning. The input features are processed through three branches before concatenation and ReLU activation. The first branch consists of a 1×1 pointwise convolution followed by a BN layer. The second branch is composed of a 3×3 deepwise convolution followed by a BN layer, and this branch is replicated three times in this study. The third branch consists of a BN layer. After concatenation and ReLU activation, the features are input into two branches. One branch consists of a 1×1 pointwise convolution followed by a BN layer, which can be replicated multiple times, and the other branch consists of a BN layer. Finally, the features are concatenated and activated with ReLU before being output. During the inference phase, a simpler structure is employed to enhance efficiency. The features only need to pass through a 3×3 deepwise convolution followed by a BN layer and a ReLU activation function. Subsequently, they go through a 1×1 pointwise convolution, again followed by a BN layer and a ReLU activation function. This structure effectively reduces the model’s parameter count while enhancing feature extraction capabilities, as shown in **Figure 8**.

#### 2.5.2 GhostConv

**Figure 9:**
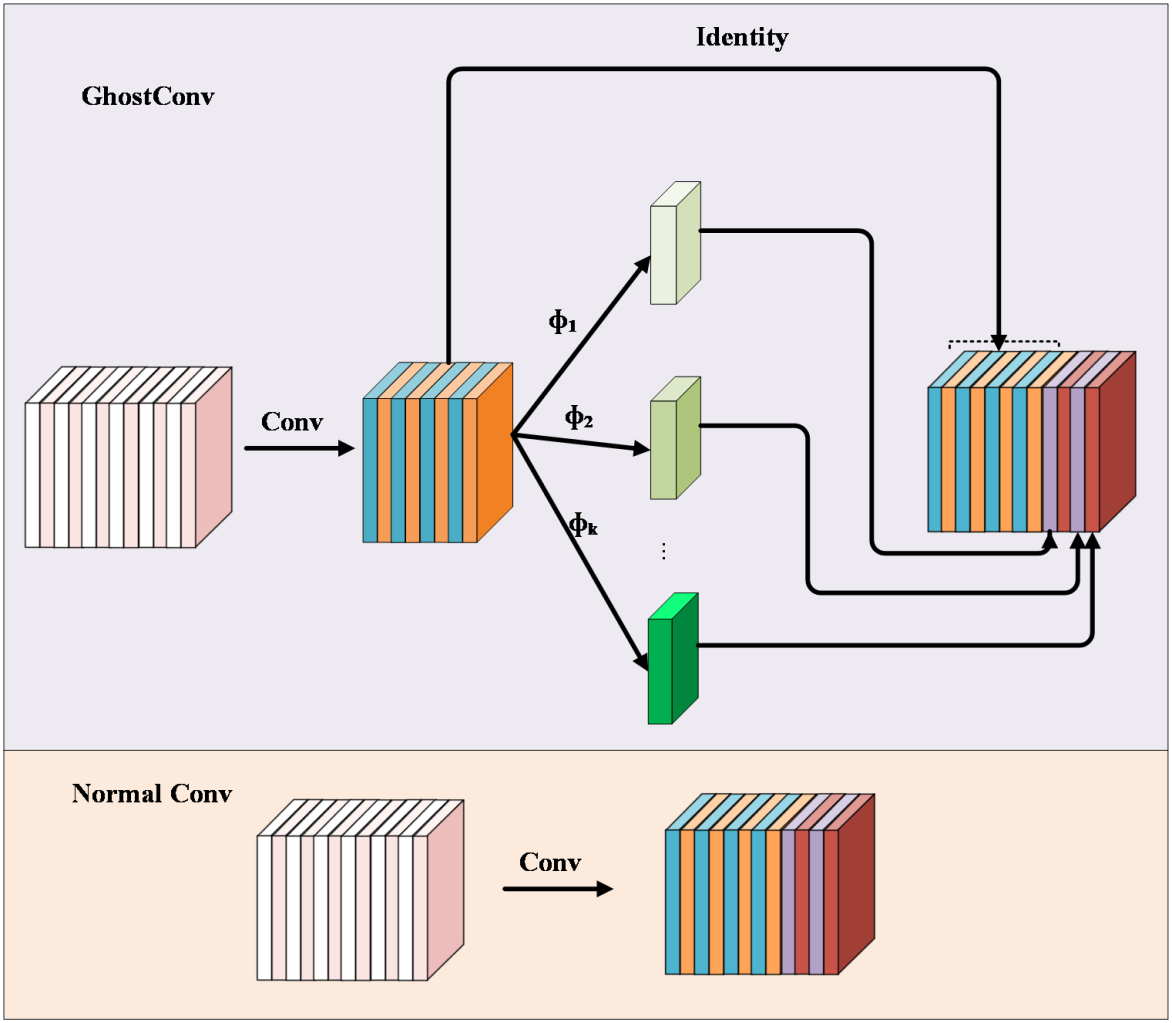
GhostConv.

The conventional approach to feature extraction typically involves using multiple convolutional kernels for convolutional mapping operations across all channels in the input feature map. In deep networks, stacking numerous convolutional layers increases parameter counts and computational requirements, resulting in many rich or even redundant feature maps. Simplifying the network by merely reducing computational efforts would entail sacrificing valuable features. To address this challenge, Han et al. (2020) introduced GhostConv, a novel approach that combines conventional convolutional operations for extracting rich feature information with cost-effective linear transformation operations to generate redundant features. This method effectively reduces the computational resources required by the model while maintaining a straightforward and easy-to-implement design. The GhostConv network structure, depicted in **Figure 9**, follows a distinctive two-step process: First, it executes conventional convolution with a controlled number of operations. The kernel size is 5×5. Second, it uses the intrinsic feature maps from the convolution to perform simple linear operations, generating additional feature maps. These feature maps from both steps are then concatenated to form a new output. This innovative design significantly reduces the network’s parameter count without sacrificing crucial image features, thereby enhancing the model’s detection speed.

#### 2.5.3 Backbone

**Figure 10:**
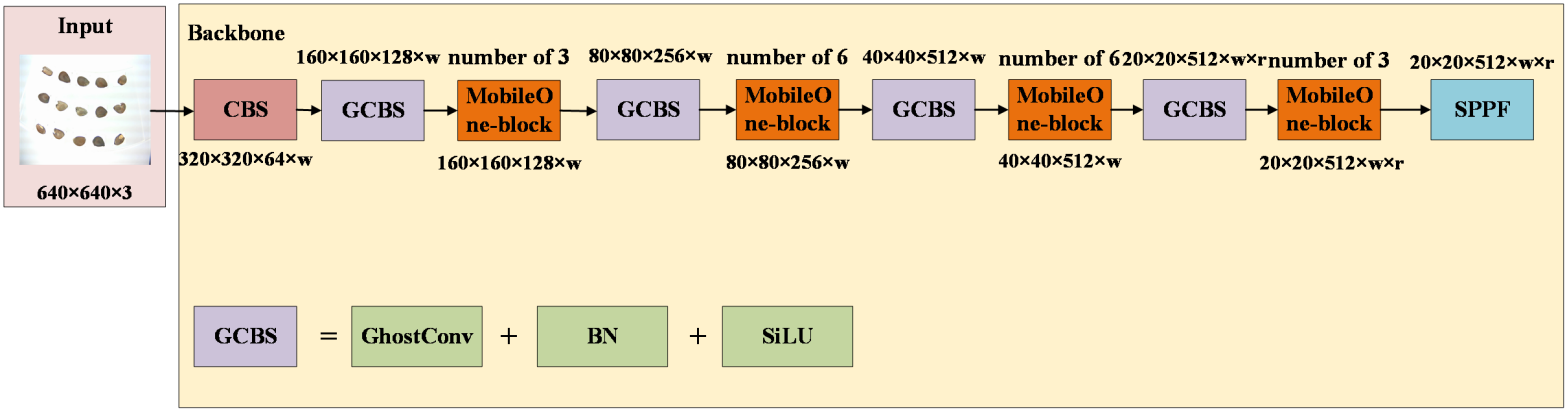
Backbone of Light-YOLO.

The primary objective of this study is to achieve rapid and accurate prediction of the location and category information for 15 cottonseeds in a given cottonseed image. Recognizing the potential of integrating the MobileOne-block and GhostConv to expedite model convergence without compromising detection performance, we aim to optimize the YOLOV8 backbone by incorporating these enhancements. The enhanced YOLOV8 backbone is depicted in **Figure 10**. In this optimization, the input image of multiple cottonseeds maintains a size of 640 × 640 pixels. The CBS module, integral to the YOLOV8 backbone, is replaced with the GCBS module, which comprises GhostConv, Batch Normalization, and SiLU. Additionally, the C2F module is substituted with the MobileOne-block module, maintaining an equivalent number of modules. The processed image then passes through the SPPF module for the final output. Similar to YOLOv8, the parameters “w” and “r” in the figure determine the network’s parameter count, corresponding to different Light-YOLO versions. Additionally, the input image size for Light-YOLO is the same as that for YOLOV8, which is 640 pixels × 640 pixels.

#### 2.5.4 YOLO head

**Figure 11:**
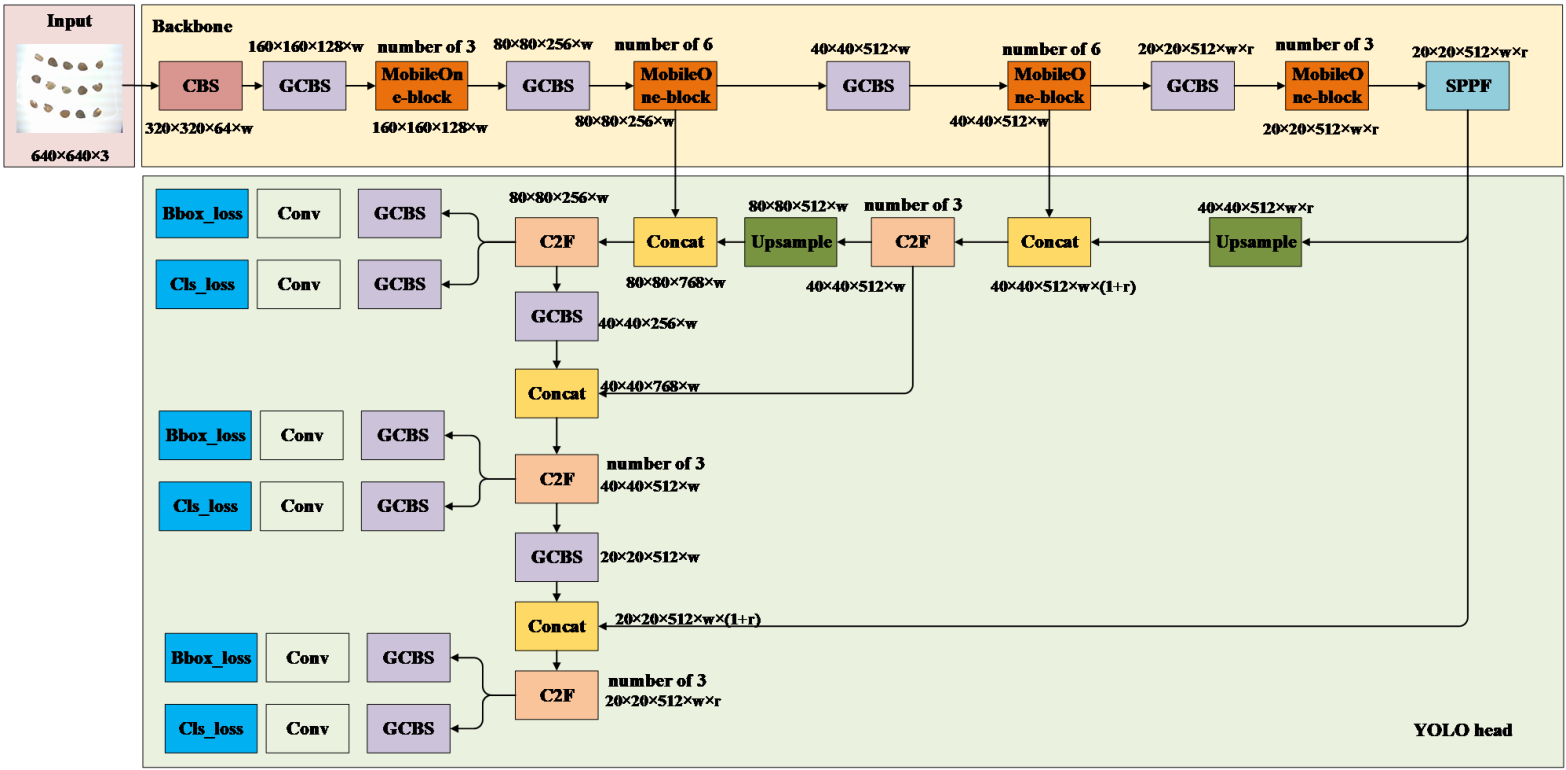
Light-YOLO.

The YOLO head plays a pivotal role in detecting category and location information for cottonseeds. In this study, considering that the C2F module enhances the gradient information of the network, it is retained in the YOLO head. However, we have replaced all CBS modules in the YOLO head of YOLOv8 with the more lightweight GCBS modules, while keeping other components unchanged, as illustrated in **Figure 11**. The YOLO head continues to extract features at three different scales (80×80, 40×40, and 20×20) from the backbone, facilitating the fusion of high and low-level features for the detection of targets of various sizes. Similar to YOLOV8, the Light-YOLO network outputs categories as “intact” and “broken,” with position outputs representing the coordinates of the 15 cottonseeds in the image. The loss function of Light-YOLO remains consistent with that of YOLOV8.

### 2.6 Indicators for model evaluation

Evaluation metrics for object detection play a crucial role in assessing the performance of detection models. They provide insights into the model’s effectiveness in identifying and localizing cottonseeds in an image. These metrics are instrumental in analyzing and comparing performance variations across different models (Li et al., 2023b). Commonly used evaluation metrics for object detection algorithms encompass Accuracy (Acc), Precision (P), Recall (R), and mAP (mean Average Precision). Accuracy gauges the overall discriminative performance of a model by determining the ratio of correctly predicted target instances to the total number of instances. Precision reveals the proportion of samples predicted as positive instances that are predicted correctly, providing insights into the model’s ability to avoid false positives. Recall represents the proportion of positive samples that the model successfully identified out of the total positive samples, reflecting the model’s proficiency in avoiding false negatives. mAP involves the calculation of mean average precision for each category, subsequently averaged across all categories, thereby delivering a comprehensive assessment of the model’s detection performance across multiple categories. This study uses these evaluation metrics to assess the effectiveness and accuracy of the proposed model in detecting intact and broken cottonseeds. The formulas for these metrics are as Formulas 7–11:


(7) $$Acc=\frac{TP+TN}{TP+TN+FP+FN}$$

(8) $$P=\frac{TP}{TP+FP}$$

(9) $$R=\frac{TP}{TP+FN}$$

(10) $$AP=\int_{0}^{1}P(R)\;dR$$

(11) $$mAP=\frac{AP_{1}+AP_{2}+\cdots +AP_{n}}{n}$$

TP denote the number of positive samples correctly classified as positive, TN denote the number of negative samples correctly classified as negative, FN denote the number of positive samples incorrectly classified as negative, FP denote the number of negative samples incorrectly classified as positive, and n represent the total number of categories. In this study, positive samples were defined as cottonseeds labeled “broken”, while negative samples were labeled as “intact”.

### 2.7 Online nondestructive detection technique and device

#### 2.7.1 Hardware of the device

**Figure 12:**
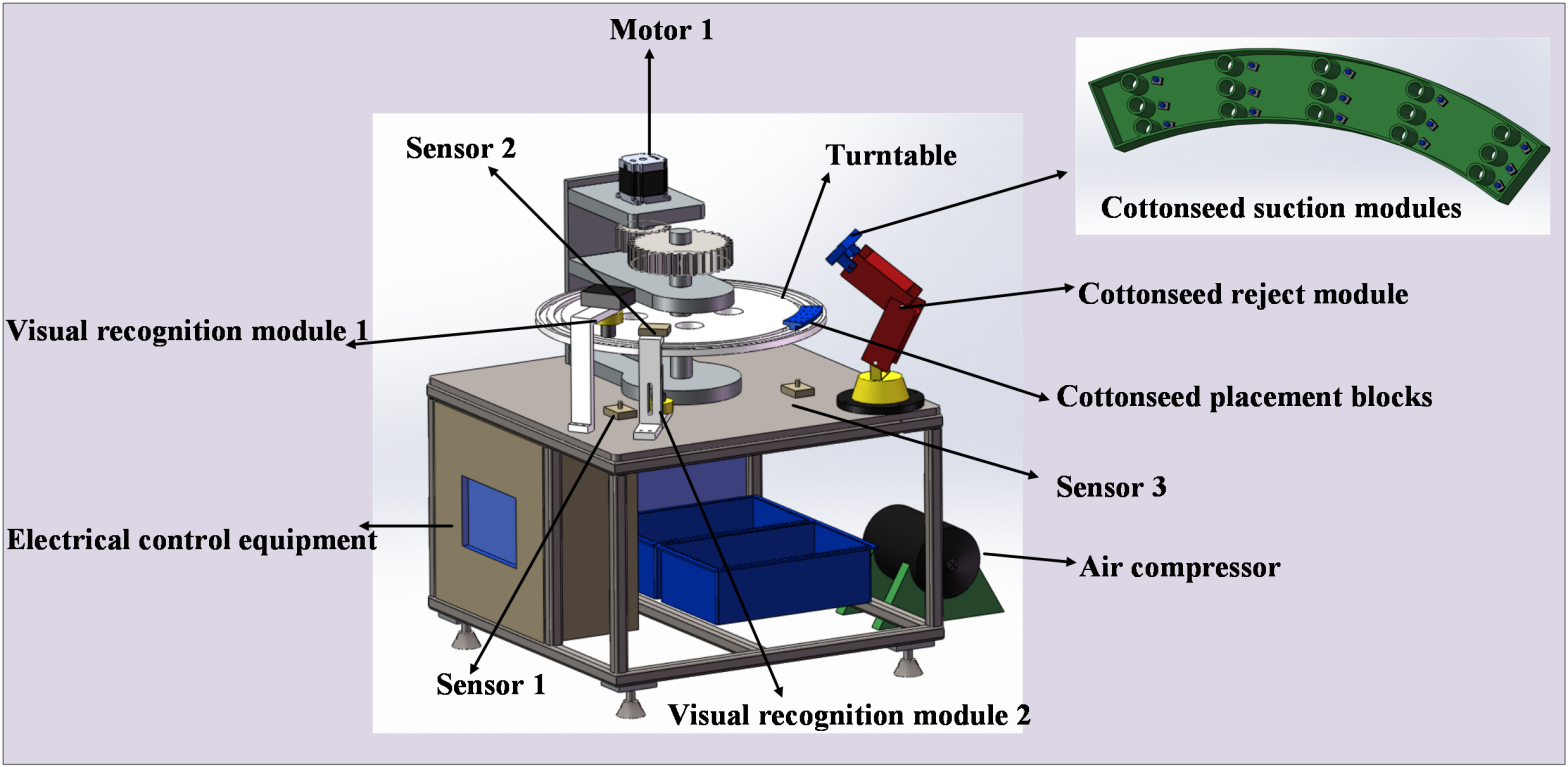
Online detection and sorting device.

In this study, we designed an online detection and sorting device tailored to the characteristics of cottonseeds, as illustrated in **Figure 12**. The device consists of electrical control equipment, a turntable, a cottonseed placement block, two visual recognition modules, a cottonseed rejection module, a motor, and various other mechanical components. The central element of the electrical control equipment is an S7–200 PLC controller, supplemented by sensors and associated control circuits. The sensor model employed is CR-10P photoelectric type sensors. The visual recognition module encompasses a camera and a light source of the same type as detailed in Chapter 2.2. The cottonseed reject module comprises a cottonseed suction module, which is equipped with a solenoid valve, a robotic arm, and an air compressor. The cottonseed suction module employs a total of 15 suction lifting groups to efficiently eliminate broken cottonseeds. The air compressor extracts air from the cottonseed suction module, creating a pressure differential, while the robotic arm facilitates the movement and operation of the cottonseed reject module. The motor in the vision, along with other mechanical modules, supplies power and hardware support for the transportation of cottonseeds.

#### 2.7.2 Software for online detection of cottonseed breakage

**Figure 13:**
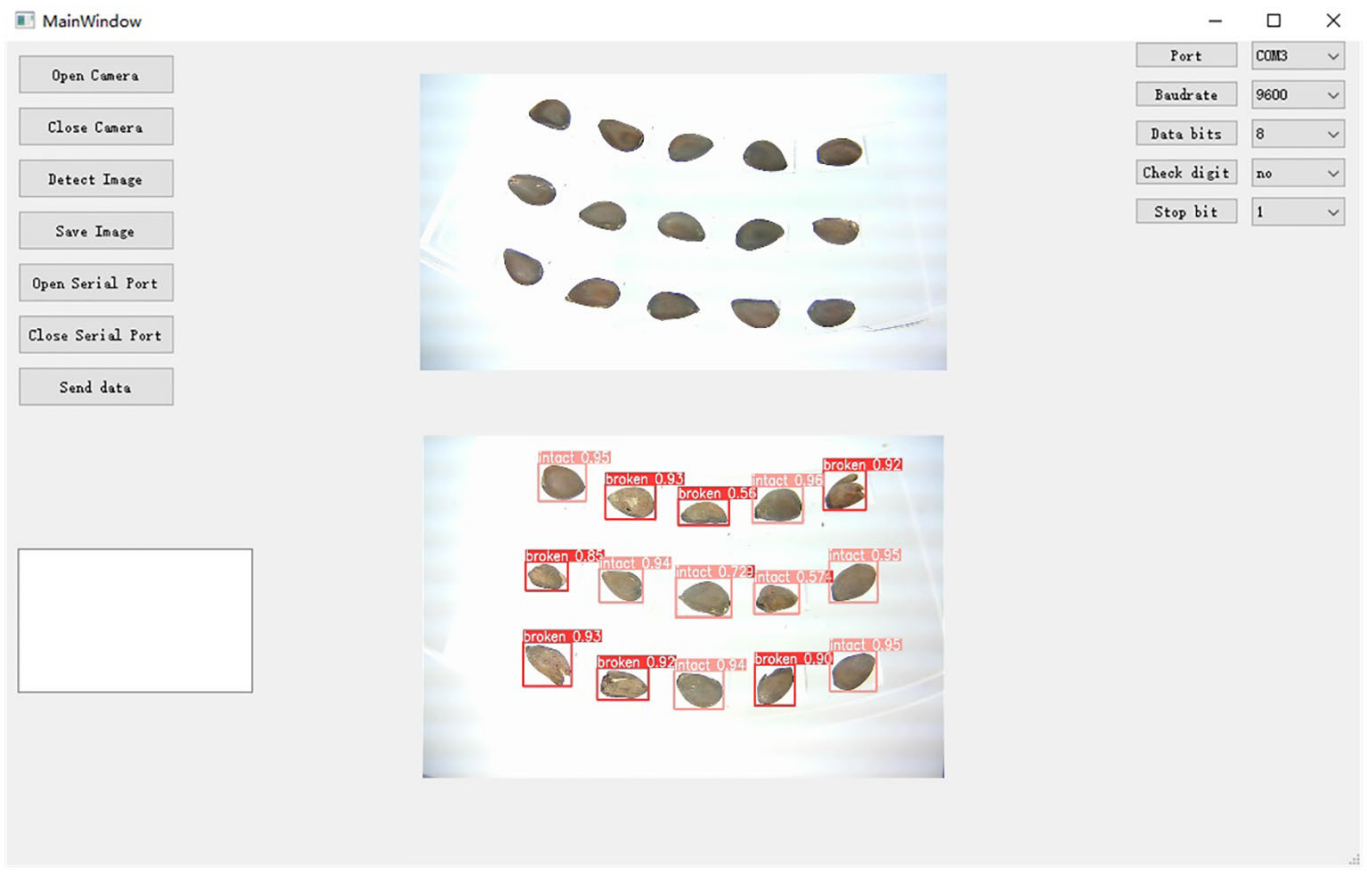
Online cottonseed breakage detection software.

To better suit the cottonseed inspection task, this study developed an online inspection software system for cottonseed breakage information using Qt, as illustrated in **Figure 13**. The software’s key functionalities include camera activation and deactivation, image saving, detection model invocation, display of the original picture and detection results, as well as communication parameter configuration. The core operations involve the activation of two cameras, the invocation of the detection model, and communication with the lower computer. Camera activation utilizes the VideoCapture API of OpenCV to manage the camera’s on/off state efficiently. For operational efficiency, the detection software exclusively displays the cottonseed images captured on the front side. The detection model is invoked by loading the torchscript.pt file using the C++ version of libtorch. Communication with the lower PLC is facilitated through Qt’s Serial Port class, enabling the adjustment of communication parameters like baud rate, data bit parity, and stop bit. Chapter 2.2 also used this detection software to acquire cottonseed images.

#### 2.7.3 Control system for online detection device

**Figure 14:**
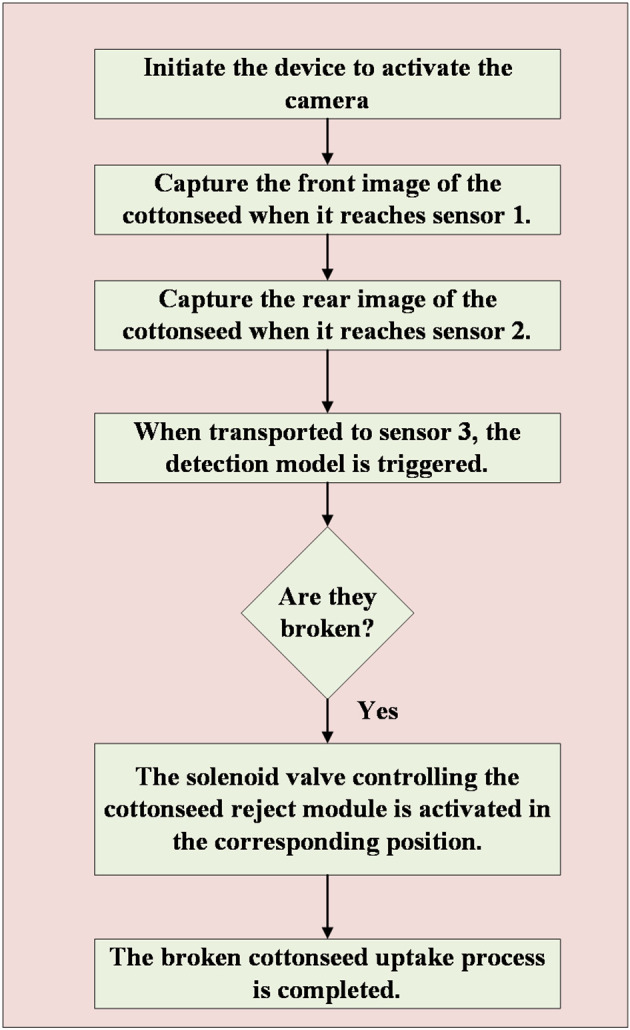
The workflow of the online detection device.

**Figure 14** illustrates the workflow of the device employed in this study. The process begins with the placement of 15 cottonseeds in the cottonseed placement block, followed by the activation of the device, which in turn activates both cameras in the detection software. Motor 1 drives the turntable to rotate, and when the cottonseed placement block reaches visual recognition module 1 (at the position of sensor 1), motor 1 pauses its rotation for 0.5s. Simultaneously, the PLC sends a signal to the detection software, prompting the camera to perform front image acquisition of the cottonseed and save it. The motor then resumes operation, and upon reaching sensor 2 (vision acquisition module 2), it pauses again for 0.5s. The PLC signals the detection software, which activates the camera for reverse image acquisition of the cottonseed and saves it. Subsequently, motor 1 continues its operation until the cottonseed placement block reaches the cottonseed reject module (at the sensor 3 position). At this point, motor 1 stops for 2s, and the PLC signals the host computer software, which triggers the detection model to discriminate cottonseed breakage. For each of the 15 cottonseeds, two detection results are obtained. If both results indicate intact cottonseed, then the cottonseed is classified as intact; otherwise, it is considered broken. The host computer software conveys the discrimination results to the PLC, which activates the solenoid valve in the cottonseed rejection module to remove the broken cottonseeds, completing the cottonseed sorting process.

After determining the equipment’s workflow, the control system can be developed, focusing on communication, input, output, and storage of cottonseed discrimination results. The communication mode selected is the serial port, with the online detection equipment for cottonseed breakage information having 3 input ports and 17 output ports. Discriminatory results are input into the register in a first-in-first-out (FIFO) manner.

#### 2.7.4 Validation experiments of online detection devices

To validate the performance of the device in detecting cottonseed breakage information, this study utilized 60 Xinhai-63 variety cottonseeds, including 30 broken and 30 intact cottonseeds. The discriminatory results of the device on cottonseeds were recorded to assess its detection effectiveness.

## 3 Results and discussion

### 3.1 Experimental configuration

The hardware platform for training and testing YOLOV8, Light-YOLO, and the other models consisted of an i9–13900HX at 2.2GHz with 32GB of RAM and 2TB of ROM. The graphics card employed was an NVIDIA GeForce RTX 4060 GPU with 8GB of video memory. The software system for training and testing was Windows 10. The computational platform included CUDA 11.7 and Cudnn version 8.6. The deep learning framework used was PyTorch 2.1, and the overall software environment operated on Python 3.9.

The training process for the object detection model aims to align the model’s predictions of the cottonseed’s category and location information as closely as possible with the actual results. The convergence speed of the model is substantially influenced by the hyperparameter settings during training. In this study, the following hyperparameters were used: (1) Learning rate: The initial learning rate was set to 0.01. (2) Patience: The training utilized an early stopping mechanism with a patience of 10 epochs, meaning that training would halt if the validation loss did not improve for 10 consecutive epochs. (3) Number of Epochs: The training process extended up to a maximum of 500 epochs. (4) Batch Size: The batch size, representing the number of images processed per batch, was configured as 4. (5) Optimizer: The Adam optimizer was employed to fine-tune the model parameters. These hyperparameters were carefully chosen to ensure efficient training and optimal model performance.

### 3.2 Results of various YOLOV8 versions

To rigorously evaluate the detection capabilities of various YOLOV8 versions for identifying cottonseed breakage, the study conducted performance tests using identical cottonseed datasets for the training set, validation set, and test set. The detection outcomes on the test set are presented in **Table 3**. From **Table 3**, it is evident that there is a substantial difference in the memory usage among different versions of YOLOV8, with YOLOV8n having the smallest memory footprint at 5.93MB, while YOLOV8x requires the most memory at 130MB. In terms of detection performance, YOLOV8x achieved the highest detection accuracy at 96.1%, while YOLOV8s and YOLOV8m attained the highest mAP50 (at IOU threshold 0.5) at 99.0%. For precision, YOLOV8x outperformed with a score of 93.8%, and for recall, YOLOV8x exhibited the highest value at 97.2%. A smaller memory footprint implies faster detection speed, YOLOV8m emerges as the best performer when balancing detection speed and accuracy. It achieves a mAP_50_ of 99.0%, Precision of 93.7%, Recall of 95.0%, Accuracy of 95.2%, and a model size of 49.6MB. In summary, YOLOV8m stands out as the most effective in detecting the location and category information of both broken and intact cottonseeds in cottonseed images. Consequently, this study has chosen YOLOV8m as the foundation for developing the Light-YOLO framework.

**Table 3:** Test results of various YOLOV8.

Model	P	R	mAP_50_	Acc	Model memory usage
YOLOV8n	89.6%	97.9%	98.5%	94.8%	5.93MB
YOLOV8s	90.7%	96.5%	99.0%	94.2%	21.4MB
YOLOV8m	93.7%	95.0%	99.0%	95.2%	49.6 MB
YOLOV8l	92.5%	96.5%	98.9%	95.2%	83.5 MB
YOLOV8x	93.8%	97.2%	98.9%	96.1%	130 MB

### 3.3 Results of ablation experiments

**Figure 15:**
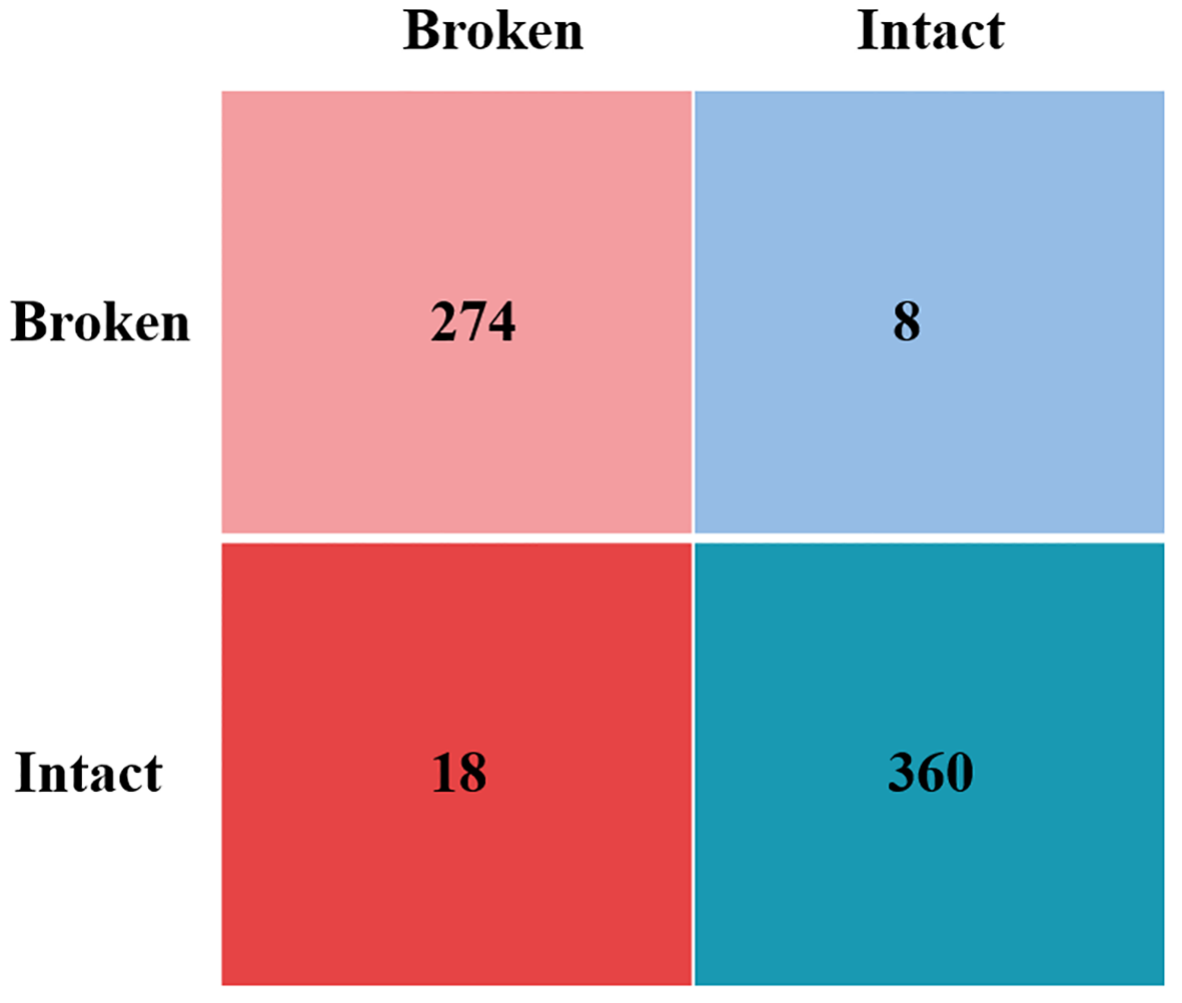
The confusion matrix for Light-YOLO.

To evaluate the efficacy of the enhancement techniques applied to YOLOV8 in this study, we conducted ablation experiments on cottonseed images sourced from the same training, test, and validation sets. For clarity, we refer to YOLOV8m with MobileOne-block as the sole improvement to the backbone as “YOLOV8m-MobileOne”, and the version incorporating GhostConv alone for enhancing both the backbone and YOLO head as “YOLOV8m-GhostConv”. **Table 4** presents the outcomes of these ablation experiments. YOLOV8m model has a maximum size of 49.6MB. Both YOLOV8m-MobileOne and YOLOV8m-GhostConv models exhibit reduced sizes compared to YOLOV8m, with 47.9MB and 45.3MB, respectively. The Light-YOLO model occupies the smallest memory footprint at 41.3MB, indicating superior running speed. For precision, Light-YOLO and YOLOV8m-MobileOne both achieve the highest at 93.8%, while YOLOV8m-GhostConv has the lowest at 91.9%. Regarding recall, the Light-YOLO model leads with 97.2%, while YOLOV8m has the lowest at 95.0%. YOLOV8m reaches the highest mAP50 at 99.0%, while the other three models achieve 98.9%. In terms of accuracy, the Light-YOLO model excels at 96.1%, while YOLOV8m-GhostConv lags at 94.8%. In conclusion, Light-YOLO not only markedly reduces the model’s parameter count but also demonstrates enhanced detection performance. This indicates that MobileOne-block and GhostConv not only decrease the number of parameters in YOLOV8 but also render the network more adept for the specific task of detecting cottonseed breakage. The confusion matrix for Light-YOLO is shown in **Figure 15**.

**Table 4:** Results of ablation experiments.

Model	P	R	mAP_50_	Acc	Model memory usage
YOLOV8m	93.7%	95.0%	99.0%	95.2%	49.6 MB
YOLOV8m-MobileOne	93.8%	95.7%	98.9%	95.5%	47.9 MB
YOLOV8m-GhostConv	91.9%	96.5%	98.9%	94.8%	45.3 MB
Light-YOLO	93.8%	97.2%	98.9%	96.1%	41.3 MB
YOLOV8m-N	92.7%	94.0%	98.6%	94.9%	49.6 MB
YOLOV8m-MobileOne-N	92.7%	95.0%	98.6%	95.2%	47.9 MB
YOLOV8m-GhostConv- N	91.2%	95.7%	98.5%	94.2%	45.3 MB
Light-YOLO-N	92.5%	96.5%	98.6%	95.5%	41.3 MB

“-N” indicates that the model is without data augmentation.

Additionally, this study compared the detection performance of four models on broken cottonseeds without using data augmentation. As shown in **Table 4**, the Precision, Recall, mAP50, and Accuracy of YOLOv8, YOLOv8m-MobileOne, YOLOv8m-GhostConv, and Light-YOLO all decreased in the absence of data augmentation. Specifically, Light-YOLO’s P, R, mAP50, and Acc decreased by 0.7%, 0.7%, 0.3%, and 0.6%, respectively. These results indicate that the data augmentation algorithms used in this study enhance the models’ detection performance.

**Figure 16:**
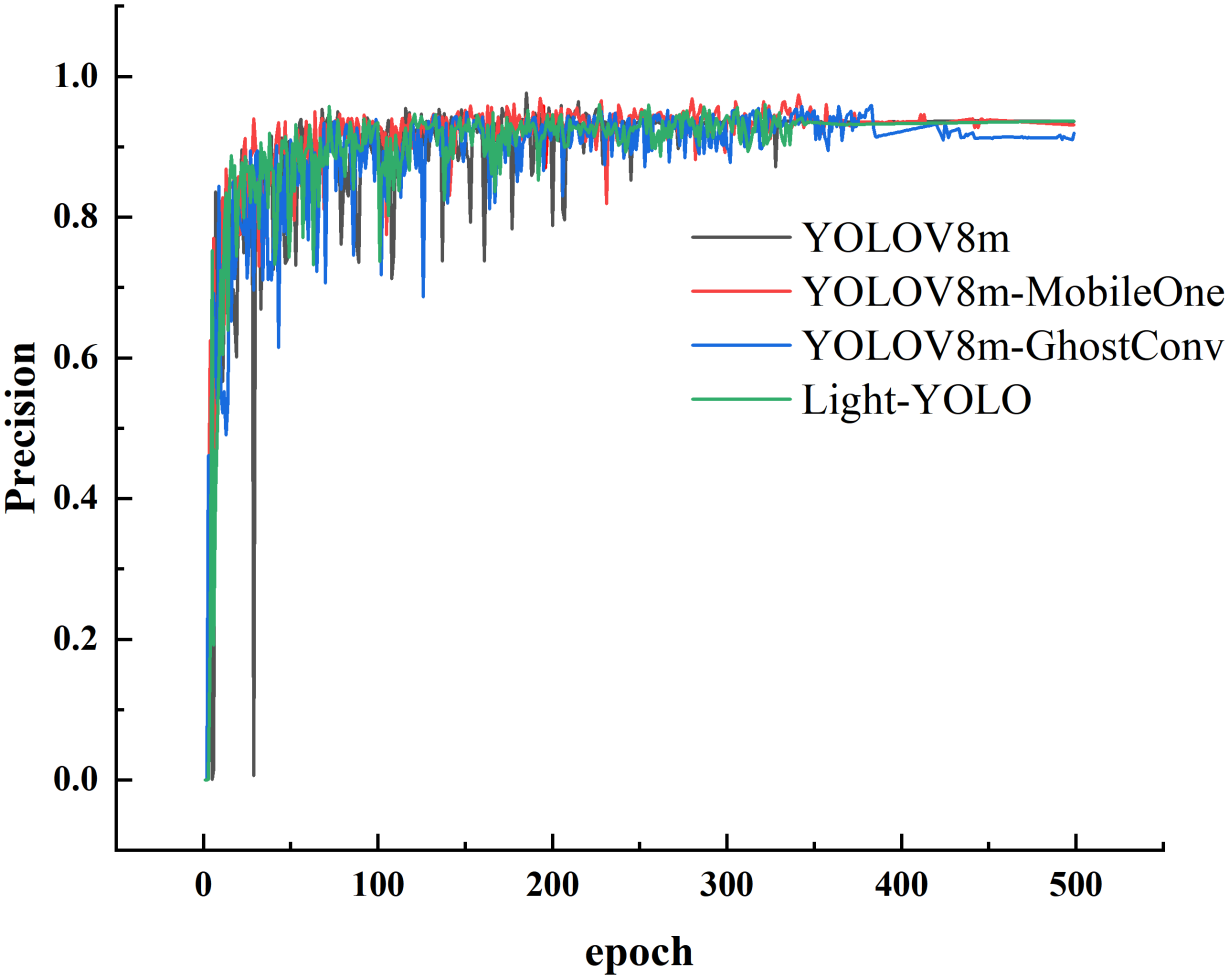
Precision change curve during training.

**Figure 16** depicts the precision change curves throughout the training process of the four models. It is evident that after 350 epochs of training, the precision starts to stabilize for all four models, signifying that they have reached convergence. Notably, after stabilization, the precision of YOLOV8m-GhostConv is the lowest, while the other three models exhibit very similar precision values. This consistency aligns with the results observed in the test set as presented in **Table 4**.

### 3.4 Comparative performance analysis against alternative models

The experimental results presented above primarily focus on comparing the detection performance of Light-YOLO and YOLOV8 regarding cottonseed category and location information in cottonseed images. However, within the realm of real-time object detection, prominent alternatives include YOLOV5 and YOLOV7. To further validate the detection capabilities of Light-YOLO for cottonseed breakage information, we conducted a comparative analysis involving Light-YOLO, YOLOV5, and YOLOV7. The results of this comparative study are summarized in **Table 5**.

**Table 5:** Comparison results with other models.

Model	P	R	mAP_50_	Acc	Model memory usage
YOLOV5	88.7%	95.0%	97.6%	92.7%	54.3 MB
YOLOV7	90.0%	95.7%	98.2%	93.6%	47.4 MB
Light-YOLO	93.8%	97.2%	98.9%	96.1%	41.3 MB

**Figure 17:**
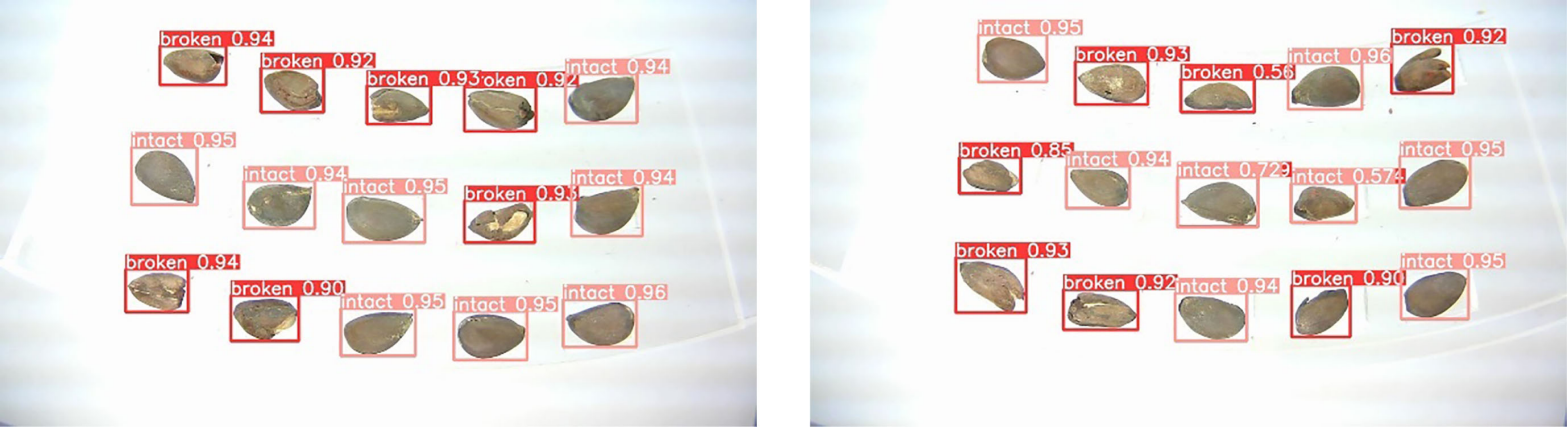
Detection results of Light-YOLO.

Comparing the detection performance of Light-YOLO with YOLOV5 and YOLOV7 for cottonseed breakage, YOLOV5 achieves a precision, recall, mAP50, and Acc of 88.7%, 95.0%, 97.6%, and 92.7%, respectively, with a model size of 54.3 MB. For YOLOV7, precision, recall, mAP50, and Acc are 90.0%, 95.7%, 98.2%, and 93.6%, respectively, with a model size of 47.4 MB. Light-YOLO outperforms both YOLOV5 and YOLOV7 in terms of detection performance and speed, with YOLOV7 slightly surpassing YOLOV5. The detection results for cottonseed images using Light-YOLO are depicted in **Figure 17**.

### 3.5 Results of online detection device

After integrating the Light-YOLO model into the cottonseed breakage online detection software, we evaluated its performance using 60 cottonseeds, divided randomly into four groups of 15. The number of cottonseeds correctly identified by the cottonseed reject module and those left on the disk were tallied. Results revealed that 6 out of 30 broken cottonseeds were mistakenly classified as intact, while 2 out of 30 intact cottonseeds were erroneously identified as broken. The total misclassification count was 8, resulting in detection accuracy of 86.7%, 80.0% for broken cottonseeds, and 93.3% for intact cottonseeds. This underscores the effectiveness and feasibility of the online detection device, providing valuable insights for subsequent research and development.

### 3.6 Discussion

To investigate an efficient technology for detecting broken cottonseeds, this study devised a collection device capable of capturing front and back images of multiple cottonseeds, tailored to the characteristics of cottonseeds. This led to the design of an online detection device for cottonseed breakage, accompanied by the development of online detection software and a control system. To enhance the detection performance of the device, we improved the backbone and YOLO head of YOLOV8 by incorporating lighter-weight MobileOne-block and GhostConv, resulting in the Light-YOLO model, optimized for online detection of cottonseed breakage. This study then conducted online experiments on the developed device to validate this approach.

Previous research has explored various techniques for detecting cottonseed breakage. Shuhua et al. (2015) employed a color sorting technique, achieving a detection accuracy of 97.8%. Gao et al. (2018) utilized spectral imaging, reporting a detection accuracy of 91.50%. Wang et al. (2023) utilized a high-speed camera, reaching a detection accuracy of 99%. Zhang et al. (2022) applied air-coupled ultrasound with a sound-to-image encoding technique, obtaining a recognition accuracy of 90.7%. In comparison, the detection technique in this study proves to be more cost-effective, quicker, and efficient, capable of simultaneously detecting 15 cottonseeds. While Liu et al. (2022) managed to detect multiple cottonseeds, their methods were limited to capturing single-side information and remained in the laboratory stage. Similar limitations were observed in the work of Du et al. (2023). In contrast, our study successfully captured both front and back images of cottonseeds, aligning more closely with the practical requirements of the cottonseed industry. This approach holds promising potential for direct application in industrial settings.

## 4 Conclusions

In response to the current lack of rapid detection techniques and methods for broken cottonseeds, this study developed an image acquisition device capable of capturing images of 15 cottonseeds simultaneously, based on the characteristics of cottonseeds. We constructed Light-YOLO, an improved model based on YOLOV8, to detect broken information in cottonseeds. Furthermore, we developed the software, hardware, and control systems for an online detection device and conducted online tests. The conclusions are as follows:

(1) MobileOne-block and GhostConv are employed to improve the backbone and YOLO-Head of YOLOV8m, resulting in Light-YOLO. The detection metrics for Light-YOLO, including precision, recall, mAP50, and accuracy for cottonseed breakage information, are 93.8%, 97.2%, 98.9%, and 96.1%, respectively, with a model size of only 41.3 MB.
(2) Ablation experiments indicate that using MobileOne-block and GhostConv alone to improve the backbone and YOLO-Head of YOLOV8m only accelerates the detection speed without enhancing the detection accuracy for broken cottonseeds. Additionally, augmenting the cottonseed image data improves the model’s detection performance.
(3) To further validate the online detection device’s performance, we tested it with 60 cottonseeds, achieving a detection accuracy of 86.7%. This underscores the effectiveness of our technology and methodology, providing crucial technical support for the efficient and rapid detection of cottonseed breakage information, with the potential for direct application in the cottonseed industry.

## Data Availability

The datasets presented in this article are not readily available because we will continue to use this data in subsequent research. Requests to access the datasets should be directed to HZ, Zhanghz_taru@163.com.
